# Supplementary material for: Different Pathogenicity and Transmissibility of Goose-Origin H5N6 Avian Influenza Viruses in Chickens
Source: Viruses. 2019 Jul 4;11(7):612. doi: 10.3390/v11070612 (PMC6669512; doi:10.3390/v11070612)
Supplement: Supplementary file 1 [file viruses-11-00612-s001.pdf]

## Supplementary information

**Table S1.** Summary of GS144 and GS148 Potential glycosylation site

| Segments | Amino acid position | Virus | |
|---|---|---|---|
| | | **GS144** | **GS148** |
| | Stalk | | |
| HA1 | 26 | NNST | NNST |
| | 27 | NSTE | NSTE |
| | 39 | NVTV | NVTV |
| | 181 | NNTN | NNTN |
| | 302 | NSSM | NSSM |
| HA2 | 500 | NGTY | NGTY |
| | 559 | NGSL | NGSL |
| NA | 59 | NITN | NITN |
| | 146 | NGTI | NGTI |
| | 190 | NASA | NASA |
| | 391 | NWSG | NWSG |
| | 402 | NWSG | NWSG |

[a] For an asparagine (N) to be glycosylated, it requires the context of the amino acid pattern N(X)S/T, where X can be any amino acid (X cannot be Proline), followed by a Serine (S) or Threonine (T).

**Table S2.** Amino-acid differences between AIVs GS144 and GS148

| Gene | Amino acid position | GS144 | GS148 |
|---|---|---|---|
| PB2 | 81 | T | I |
| | 109 | V | I |
| | 117 | P | T |
| | 125 | L | V |
| | 409 | R | C |
| | 490 | K | N |
| | 590 | W | G |
| PB1 | 178 | I | V |
| HA (H5 - numbering) | 179 | I | T |
| | 190 | M | V |
| | 209 | P | S |
| | 242 | T | A |
| | 243 | N | T |
| | 257 | Y | F |
| | 386 | E | Q |
| NP | 50 | S | G |
| | 61 | I | M |
| | 84 | N | S |
| | 408 | I | V |
| NA (N6 - numbering) | 27 | T | A |
| | 76 | M | V |
| | 137 | S | G |
| | 174 | V | I |
| | 387 | T | A |
| M | 261 | Y | H |
| NS1 | 100 | I | L |

**Table S3.** Summary of signature amino acid Mutations in GS144 and GS148

| Virus | Segment | Summary of Amino acid residues Mutation | | | | | | | |
|---|---|---|---|---|---|---|---|---|---|
| | | PB1 | HA(H5) | NP | | | M1 | | NS1 |
| | | L13P | D190E | M61I | S319N | L479F | N30D | T215A | D92E |
| GS144 | | P | E | I | N | F | D | A | E |
| GS148 | | P | E | M | N | F | D | A | E |

[a] Amino acids sites HA segment was located by taking A/goose/Guangdong/1/1996 (H5N1)
